# Intubation of the Larynx

**Published:** 1887-06

**Authors:** Henry D. Ingraham

**Affiliations:** Professor of Diseases of Women and Children, Medical Department of Niagara University, Gynœcologist to the Sisters of Charity Hospital


### *INTUBATION OF THE LARYNX.**

By HENRY D. INGRAHAM, M. D.,
Professor of Diseases of Women and Children, Medical Department of Niagara University,
Gynœcologist to the Sisters of Charity Hospital.

The first intelligent attempt ever made to tube the larynx, was that of M. Bouchut, of Paris, in 1858. This gentleman appeared before the Academy of Medicine in that city, and advocated a method of intubation of the larynx in cases of laryngeal stenosis and dyspnœa, as a substitute for tracheotomy. He proposed the use of cylindrical tubes three-fourths of an inch to one inch long, and one-fourth of an inch or a little more in diameter, introduced on the point of a hollow sound and the tube allowed to rest on the vocal cords. A silk thread was attached to the tubes to facilitate their withdrawal when necessary. He recited seven cases—five of them had died, and two recovered after having tracheotomy performed. Notwithstanding the unfavorable result of his experience, Bouchut in commending tubage declaimed against tracheotomy, which aroused the antagonism and opposition of Trosseau, its strongest advocate, to such an extent that the new procedure failed, both in theory and practice, to commend itself to the profession.

*Read at the annual meeting of the Alumni Association, Medical Department, Niagara University, April, 1887.

Bouchut demonstrated but one fact, viz., that the larynx will tolerate a tube. A committee appointed by the Academy of Medicine to investigate the method reported that intubation of the larynx was impracticable.

It remained for Dr. Joseph O'Dwyer, of New York, to perfect a method of intubation, which is not only ingenious but practicable, and without doubt it has been the means of saving many lives.

Dr. O'Dwyer was ignorant of the experiments of Bouchut or the *dictum* of the Paris Academy, and he arrived at success after long, tedious and untiring efforts. He had perfected the instrument so completely, had tested the matter so thoroughly before bringing it to the notice of the profession, that there was but little opportunity left for improvement. Even after five years, experimenting on the cadaver and practice upon the living, and when he had accomplished so much he had not the assurance to justify himself in bringing his most wonderful discovery before the profession until he had given it farther trial, and doubtless would not have done so as soon as he did, had it not been for his friends.

All of you who have been in practice any length of time know too well the terrors of membranous or diphtheritic croup. You dread to be called to see a case, because the sufferings of the patient are fearful to behold, and the result is usually fatal. Sometimes little by little, but often quite rapidly, the laryngeal stenosis increases, and soon the patient begins to experience the want of air.

He throws his head back as far as possible in order to increase the capacity of the trachea, the chest is heaved violently at each effort to inspire, and the larynx is depressed forcibly towards the sternum, while the abdominal muscles co-operate energetically in expiration. The face is heavy and anxious, the eyes are dull, the lips livid, the skin dry, the extremities cold, or a clammy sweat breaks out upon the surface. The respiration is hurried, unequal and irregular, while the pulse is very frequent and feeble.

The child throws itself about and puts its hands to its throat as though to tear away some obstacle to the admission of air, while helpless, hopeless agony is depicted on its countenance.

You know that the false membrane is occluding the larynx. You also know that relief is only possible by the removal of the obstruction to the passage of air. The mother appeals to you to save her child, or, if that cannot be done, to at least do something to relieve its suffering. Perhaps tracheotomy has been proposed, but the friends object. They may say as I once heard a German upon whose child it was proposed to operate, that it might do for dogs, but he was not going to have his child's throat cut; if he must die, he was not going to have him tortured.

With the great objections there is against tracheotomy, and the poor results obtained, no one feels like urging the operation very strongly.

Doubtless you are all familiar with the instruments of Dr. O'Dwyer and the mode of using them at least in theory, if not in practice.

I will not occupy your time with a detailed description of the operation, but will call your attention to some particular points. It is necessary to have the child's arms secured, and the best way is to fold a sheet lengthwise about a foot wide and wrap it around the body, binding the arms securely to its side. Some one holds the child, and another assistant holds the child's head in the natural position, not thrown back. The mouth-gag is now introduced between the teeth, and it may be necessary to have this held, as some children throw it out even when carefully placed. You then pass the index finger of the left hand over the tongue, hook up the epiglottis, and feel the opening of the larynx. The handle of the applicator should be held near the child's sternum until the tube has reached the pharyngeal wall, when the handle is rapidly elevated and the tube directed downward and forward along the finger into the larynx. I believe it is better to pass the tube by the side of the finger instead of in front of it, as I formerly did, because in young children the epiglottis is flaccid, and the end of the tube may push it down and prevent the introduction of the tube. I think this has happened to me. But when you reach the end of the finger, you pass the tube directly *under* it, not over nor by the end of it. This is an important point.

When the tube is removed, the extractor is introduced under the finger in exactly the same manner. If the tube has been inserted into the larynx, respiration becomes easier. It is well to wait a minute or two before cutting the thread and withdrawing it, simply to be sure that the tube is in proper position.

There are several modifications of the O'Dwyer tubes, but I have no personal knowledge of any of them.

The relative value of tracheotomy and intubation as a means of saving life is shown by the following statistics:

Dr. A. Jacobi, of New York, who probably has had a larger experience than any other physician or surgeon in this country, reports that in over 400 tracheotomies that he has performed, only about a total of 12 per cent. have recovered.

Prof. Jacobi's statistics are more favorable than the average result, although I am aware that Drs. J. H. Ripley and Fred. Lange, of New York, report in 66 cases 33⅓ per cent. of recoveries.

Dr. Brush, the gentleman who first brought before the profession Dr. O'Dwyer's method, told me last February that he had intubated in eight cases, and all terminated fatally.

In a letter received from Dr. O'Dwyer, written April 8th, he says: "I have practiced intubation of the larynx since the beginning of my experiments in 1880, in 134 cases of croup, membranous or diphtheritic, with 26 recoveries. During the first three years of this time, or while using very imperfect forms of tubes, I had no recovery. I have operated in private practice now 69 times, with 17 recoveries." Thus it appears that Dr. O'Dwyer has had 19.4 per cent. of recoveries, including all of his cases, while in private practice, and since the use of his more perfect tubes, the recoveries have been 24.6 per cent. He also writes me that he has been called to operate upon five other cases of diphtheritic croup where he did not think it necessary, and the patients recovered without any operation.

Dr. Waxham reports (*Chicago Medical Journal and Examiner*, August, 1886,) that the record of recoveries in Chicago after tracheotomy has been 18.95 per cent., while that of intubation has been 27.71 per cent.

I have had four cases of intubation of the larynx, and although they all terminated fatally, there is no doubt in my mind but that one of them would have recovered had the child's mother obeyed the instructions given her, although the condition of the patient at the time of the introduction of the tube was very critical, and it was not expected that she could last long unless relief was afforded her. Upon the introduction of the tube, relief was almost immediate, and the child continued to improve for three days, when she was suddenly seized with a severe paroxysm of coughing and strangling, caused, as I believe, by drinking a glass of water, and suddenly expired.

In the other cases it was not expected when the tubes were introduced that recovery was possible, but in two of these cases the relief from the distressing dyspnœa was more than sufficient to justify the operation, and the parents of the children so expressed themselves.

In one case I was obliged to use too small a tube, the proper size being in the throat of another child at the time. The tube was coughed up and re-inserted three times without any apparent injury to the child.

It being true that the percentage of recoveries is greater from intubation than tracheotomy, and certainly there can be no doubt upon this point, it is much the preferable operation of the two. Anything which saves the life of the patient, or any procedure which saves more lives than another, is the proper one to adopt.

But besides the more favorable results, there are many other reasons which recommend intubation over tracheotomy.

Chief among these are that there is no objection upon the part of the parents, or, at least, but little; and after it has been proposed and explained, the parents themselves usually ask for the introduction of the tube, if for any reason it be delayed.

In most cases, after the operation the relief is marvelous. Physicians of many years' experience say that they have never seen anything like it. The child usually expresses himself as greatly relieved, and in one case which I had, the patient said, soon after the introduction of the tube, "You are good doctors, to make me feel so much better."

If the child dies, there is no regret upon the part of the physician or friends that the operation was performed, usually only feelings of gratitude that the sufferings of the little patient have been so greatly relieved, even if his life has not been saved. Certainly, if the operation did no more than to relieve the terrible sufferings of the patient, it would, in my mind, be sufficient to fully commend it.

The tube is worn without much irritation, and expectoration occurs more readily than through the tube used in tracheotomy. The air that enters the lungs through the tube is warm and moist from its course through the upper air passages, and bronchitis or pneumonia is not as likely to occur as it is after tracheotomy.

In intubation, the operation is bloodless, and there is no open wound, which in itself may be a source of danger. It is more quickly performed, and with less danger than tracheotomy. If the patient recovers, convalescence is more rapid, the patient usually regaining the use of his voice in seven to ten days after the removal of the tube. Still another advantage is that the patient does not need the constant care of the surgeon or some skilled person, as in tracheotomy.

Although there are many reasons to recommend intubation, there are, as with everything else, several objections to it. One of these is the difficulty of introducing the tube, which in some cases, it must be confessed, is great; yet, a sufficient amount of practice upon the cadaver, and a reasonable amount of dexterity, combined with coolness, there is usually no serious difficulty in the operation. If it is difficult, it is not a sufficient reason to prevent all being done to relieve the patient that is possible. Another objection is the inability of some patients to swallow liquids; all can swallow solids and semi-solids without difficulty, and most patients can swallow liquids after a little practice without much trouble, if the nurse is careful to give it to them, at first, while lying on the side. In some cases the epiglottis does not seem to close over the tube, and then the child is unable to swallow liquids without coughing, indicating that some of the liquid has passed through the tube, and hence the dangers of lung complications are increased. Dr. O'Dwyer told me, if I understood him correctly, that he did not think there was much, if any, danger of any fluid passing through the tube in any case; that he considers it safe to let a child drink a reasonable quantity of liquid if he wishes it. His experience is vastly greater than mine, yet I cannot but believe that the first case I had would have recovered if she had not been allowed to drink all she wished. This patient was doing very well, had greatly improved, and presented every indication of recovery. She could not, however, swallow liquids without coughing, unless she lay on her side, and took only a teaspoonfnl at a time.

Contrary to strict orders, the mother gave the patient a tumblerful of water, which she drank. She immediately had a violent fit of coughing and strangling, and in less than thirty minutes was dead.

Unfortunately, croup appears to occur more frequently in families where the parents are not remarkable for intelligence, or particular to follow closely the advice of the physician, and the care and attention which the patients receive is usually none of the best. After intubation the relief is usually so great that it is often very difficult to convince the friends that the child is still very ill; that you have only given it one more opportunity to get well—that it is yet suffering from a very grave disease, with the chances of recovery much against it. They are so pleased with the changed condition of the sufferer, who a few moments ago was on the verge of suffocation, that they cannot believe what you tell them, hence the patient does not receive the care and attention which he should. When we can have an intelligent nurse—one who will carefully attend to the administration or non-administration of food and drinks, and conscientiously carry out our instructions—and when the operation can be performed dexterously by the physician, I believe the recoveries from intubation will be much greater than at present reported, and the operation come into more frequent use.

